# MEG Source Localization of Spatially Extended Generators of Epileptic Activity: Comparing Entropic and Hierarchical Bayesian Approaches

**DOI:** 10.1371/journal.pone.0055969

**Published:** 2013-02-13

**Authors:** Rasheda Arman Chowdhury, Jean Marc Lina, Eliane Kobayashi, Christophe Grova

**Affiliations:** 1 Multimodal Functional Imaging Laboratory, Biomedical Engineering Department, McGill University, Montreal, Canada; 2 Neurology and Neurosurgery Department, Montreal Neurological Institute, McGill University, Montreal, Canada; 3 Department of Electrical Engineering, Ecole de Technologie Supérieure, Montréal, Canada; 4 Centre de recherches mathématiques, Université de Montréal, Montréal, Canada; University College of London - Institute of Neurology, United Kingdom

## Abstract

Localizing the generators of epileptic activity in the brain using Electro-EncephaloGraphy (EEG) or Magneto-EncephaloGraphy (MEG) signals is of particular interest during the pre-surgical investigation of epilepsy. Epileptic discharges can be detectable from background brain activity, provided they are associated with spatially extended generators. Using realistic simulations of epileptic activity, this study evaluates the ability of distributed source localization methods to accurately estimate the location of the generators and their sensitivity to the spatial extent of such generators when using MEG data. Source localization methods based on two types of realistic models have been investigated: (i) brain activity may be modeled using cortical parcels and (ii) brain activity is assumed to be locally smooth within each parcel. A Data Driven Parcellization (DDP) method was used to segment the cortical surface into non-overlapping parcels and diffusion-based spatial priors were used to model local spatial smoothness within parcels. These models were implemented within the Maximum Entropy on the Mean (MEM) and the Hierarchical Bayesian (HB) source localization frameworks. We proposed new methods in this context and compared them with other standard ones using Monte Carlo simulations of realistic MEG data involving sources of several spatial extents and depths. Detection accuracy of each method was quantified using Receiver Operating Characteristic (ROC) analysis and localization error metrics. Our results showed that methods implemented within the MEM framework were sensitive to all spatial extents of the sources ranging from 3 cm^2^ to 30 cm^2^, whatever were the number and size of the parcels defining the model. To reach a similar level of accuracy within the HB framework, a model using parcels larger than the size of the sources should be considered.

## Introduction

Epilepsy is a neurological disorder characterized by the recurrence of clinical seizures. The state during which the seizure takes place is called the ictal state. In between the seizures, abnormal neuronal discharges, the so-called inter-ictal spikes may take place and usually occur more frequently than the seizures. They are generated by the brain without any clinical manifestations and originate partially from brain regions similar to the ones involved during the seizures, i.e., from the epileptogenic focus. Thus analysis of inter-ictal spikes is widely used as a marker of epilepsy [1]–[3]. The context of the present study is the identification and localization of the epileptogenic focus using these markers, which is crucial during pre-surgical evaluation of epilepsy surgery [4], [5].

Epileptic activity originates from abnormal excitability and synchronization of neurons. The large pyramidal neurons of the cortical layer V, which are oriented perpendicularly to the cortical surface of the brain, are the main generators of brain electro-magnetic activity. Magneto-Encephalography (MEG) measures the magnetic fields generated by the neuronal currents, using a helmet of few hundred sensors uniformly distributed around the head [6], [7]. This non-invasive modality is used to localize brain regions involved during the generation of epileptic discharges [3], [8]–[10].

The amplitude of MEG signals for physiological brain activity is expected to range from femto-Teslas to pico-Teslas. As mentioned by Huiskamp et al. [11], inter-ictal spikes are spontaneous signals that can have relatively large amplitude (∼3 pT in MEG). This implies that epileptic MEG signals are likely to arise from large spatially extended regions of active cortex [12]. A study by Mikuni et al. [13] suggested that MEG can detect epileptiform activity when a cortical area greater than 4 cm^2^ is synchronously involved. Comparing MEG spikes with Electro-CorticoGraphy (ECoG) spikes, studies performed by Oishi et al. [14] and Huiskamp et al. [11] showed that MEG sensitivity varies for different regions in the brain. As a result, not only the size of the generators matters, but their location and orientation affect the detection of the MEG epileptic activity [1], [13].

The MEG inverse problem of source localization consists in inferring the location of the generators of brain activity from signals detected outside the head [15]. Following a previous study in which we proposed source localization techniques that are sensitive to the spatial extent of the generators of epileptic activity in EEG [16], the present study aims at evaluating the performance of similar methods when applied on MEG data in this context. MEG source localization did show excellent spatial accuracy when validated using invasive studies such as ECoG [14], [17], depth electrode recordings [18], [19] and post-operative follow-up [20]. While, MEG offers an excellent temporal resolution (few milliseconds), our main objective is to propose a source localization technique that is sensitive to the spatial extent of the underlying generators.

MEG source localization is an ill-posed problem, as it admits no unique solution unless additional information is used to regularize the problem. Such regularizations consist in adding some *a priori* knowledge or constraints to the problem. For instance, Dale and Sereno [21] introduced anatomical constraints that provided prior information about the sources by fixing the position of the sources along the cortical surface in a distributed source model. This type of constraint makes the inverse problem linear. However, the problem is still under-determined due to the use of few sensors (around 300) to estimate brain activity over a large number of sources (around 4000).

In order to obtain a unique solution, additional constraints in the form of a regularization scheme are required. Minimum Norm Estimate (MNE), which chooses the minimum energy solution [22], and Low Resolution Electromagnetic Tomography (LORETA) [23], which chooses the solution with maximum spatial smoothness are among the first and still very popular regularization techniques proposed to solve this issue. In the present study, we compared two regularization schemes based on the following statistical frameworks: (1) the Maximum Entropy on the Mean (MEM) [16], [24], [25] and (2) the Hierarchical Bayesian (HB) framework [26], [27], because of their flexibility in including prior information or constraint models of different natures.

Based on the rationale of obtaining realistic constraint models describing the generators of epileptic activity, two types of spatial models have been investigated. The first one is the idea that brain activity may be modeled as organized among cortical parcels, that can be active or not, when contributing to specific activity [16], [24], [28]. The second model is an extension of the spatial smoothness constraint originally proposed in LORETA [23] but locally constrained within cortical parcels as proposed by Trujillo-Barreto et al. [29]. Clustering of the brain activity into non-overlapping cortical parcels is achieved using a Data Driven Parcellization (DDP) technique similar to the ones described in Amblard et al. (2004) [24], Lapalme et al. (2006) [28], and Grova et al. (2006) [16]. In this study we denoted P(s) the spatial clustering of the whole cortical surface at a spatial scale *s*, controlling the spatial extent and the total number of parcels.

In order to implement these above-mentioned spatial models, we proposed two new source localization methods within the MEM framework (MEM-s and CMEM-s) and one within the HB framework (COH-s). MEM-s refers to the MEM approach proposed in Grova et al. [16] at a specific clustering scale *s*, while CMEM-s refers to “Coherent”-MEM-s, introducing local spatial smoothness within each cortical parcel. On the other hand, within the HB framework, we proposed the “Coherent at scale *s*” (COH-s) localization method, modeling the covariance of the sources as a linear combination of source covariance components [27], where each component defines local spatial smoothness over a parcel of P(s). COH-s uses the same spatial model as CMEM-s and has been designed to compare MEM and HB frameworks in similar conditions. In order to assess the ability of these three methods to localize spatially extended epileptogenic generators, we evaluated them within a fully controlled environment using realistic simulations of MEG data. MEM-s, CMEM-s and COH-s were evaluated together with two HB methods, proposed in Friston et al. (2008) [27]: the independent and identically distributed sources (IID) model and the spatially coherent sources (COH) model, as implemented in the SPM8 software (http://www.fil.ion.ucl.ac.uk/spm/software/spm8). We assessed the detection accuracy of all the methods by simulating sources of several spatial extents *s_e_* ranging from ∼3 cm^2^ to 30 cm^2^, and at different cortical depths. Secondly, for the three methods (COH-s, MEM-s and CMEM-s) using the spatial model P(s), we assessed the influence of the spatial clustering scale *s* on their detection accuracy. We quantified the performance of each method using the area under the ROC curve (AUC) as an index of detection accuracy. We also considered the Mean Square Error (MSE) and minimum geodesic distance (Dmin) as localization error metrics [16].

After introducing the MEG inverse problem using a distributed source model, the definition of the two general spatial models considered in this study is provided: (i) the DDP and (ii) the local spatial smoothness. Then, the MEM framework and the implementation of MEM-s and CMEM-s methods are described, followed by the description of the HB framework and the corresponding methods (COH-s, COH and IID). The evaluation procedure of the source localization methods using realistic simulations is then introduced. Finally the results and a detailed discussion are presented.

## Materials and Methods

### MEG Inverse Solution Using Distributed Model

A distributed source model consists of a large number of dipolar sources distributed along the cortical surface. We considered the orientation of each dipole to be fixed perpendicular to the cortical surface. Using this anatomical constraint, the relationship between source amplitudes and MEG measurements is expressed by the following linear model [21]:
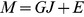
(1)where *M* is a 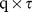 matrix of the MEG signal measured at q = 275 MEG sensors and 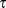 time samples. *E* models an additive measurement noise (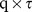 matrix). *J* is a 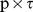 unknown matrix of the current density along the cortical surface (p∼4000: unknown dipolar moment amplitudes). *G* indicates the 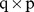 lead field matrix obtained by solving the forward problem, by estimating the contribution of each dipolar source on the sensors.

However, the inverse problem is still an ill-posed problem as the forward matrix *G* is under-determined (p>>q). There is no unique solution unless a priori model or assumptions regarding the distribution of the sources *J* are added to regularize the problem. To solve the ill-posed inverse problem, we investigated the relevance of two types of spatial models, DDP and local spatial smoothness, within two regularization frameworks (MEM and HB). In the next sections, we will describe these two spatial models before introducing their implementation within the MEM and the HB frameworks.

### Definition of Realistic Spatial Models for Spatially Extended Generators

#### Data Driven Parcellization (DDP) of the cortical surface

We first assume that brain activity can be organized into functional cortical parcels. Characterizing brain activity, assuming functional homogeneity within brain parcels has proved to be an efficient approach to analyze neuroimaging data, either in EEG/MEG [16], [28]–[31], in fMRI [32]–[34] or in multimodal fusion [35], [36].

**Figure 1:**
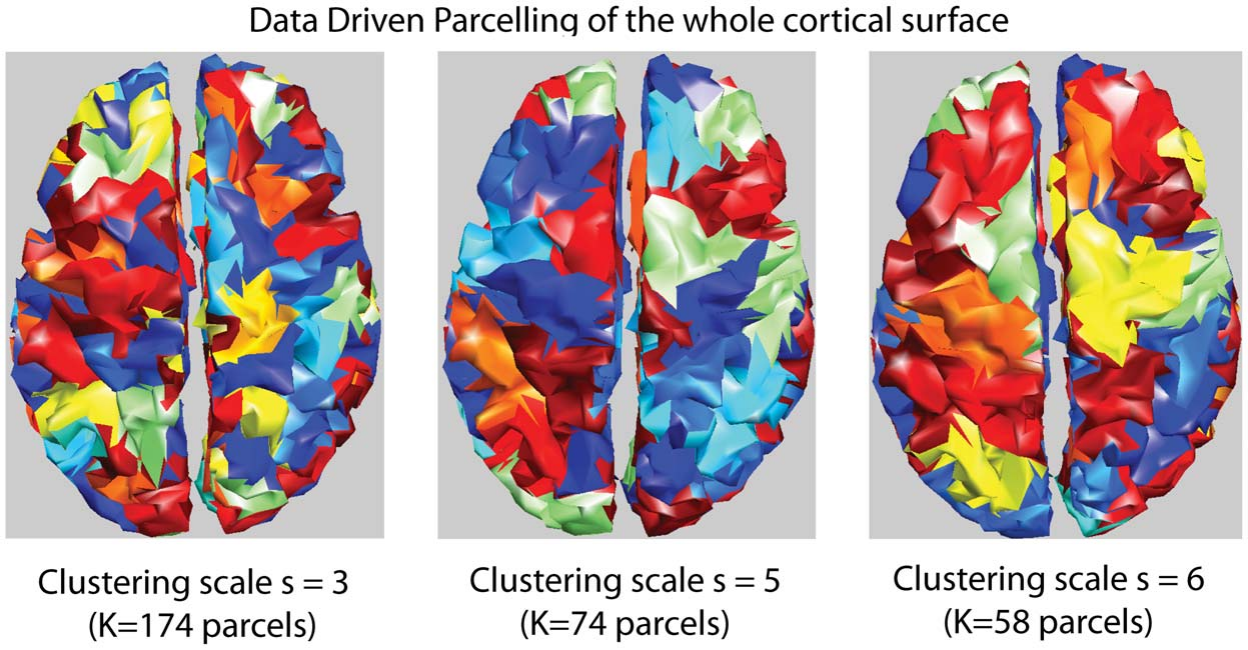
Parcellization. Examples of clustering of the cortical surface at different spatial scales s obtained using the DDP technique (each color represents one parcel).

In the present study, we proposed a Data Driven Parcellization (DDP) method performing full parceling of the tessellated cortical surface into non-overlapping parcels (see Figure 1). Such a partition at a specific spatial scale *s* is denoted by P(s). DDP consists in using partial information from the available data in order to guide this spatial clustering.

The key aspect of DDP lies in the pre-localization of the sources of brain activity using the Multivariate Source Pre-localization (MSP) method [30] followed by a region growing algorithm. MSP is a projection method that estimates a coefficient, which characterizes the possible contribution of each dipolar source to the data. A spatio-temporal extension of the MSP method is described in Appendix S1. From this extension, seed points were iteratively selected among the dipoles showing the highest MSP coefficients. Region growing around each seed points was then iterated until a given spatial neighborhood order *s*, resulting in a partition of the whole brain into K parcels. This way of choosing the seed points and parceling ensured dipoles contributing to the same underlying generator to be gathered within the same parcel, whereas dipoles contributing to distinct generators to be associated within distinct parcels. A brief description of this DDP technique is provided in Appendix S2.

Defining brain activity in terms of K parcels of functionally homogenous activity (K<<p) aims at better conditioning the under-determined inverse problem, while the inverse method will infer the local source intensity inside each parcel.

#### Local spatial smoothness model

Spatial smoothness model assumes that nearby dipoles are more likely to have similar intensities. In this context, LORETA - originally proposed by Pascual-Marqui et al. [23] - used a discrete Laplacian operator to find the solution with maximum spatial smoothness over a 3D grid.

In order to introduce local spatial smoothness over a geodesic surface, we used the diffusion-based spatial prior proposed by Harrison et al. [37]. Diffusion-based spatial priors are actually constructed using the Green’s function of the adjacency matrix defined over the geodesic cortical surface [37], [38]. Let us denote 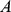 as the 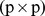 adjacency matrix of the cortical surface, where 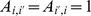, if the dipoles *i* and *i’* are distinct and directly connected on the mesh, 0 otherwise. The non-zero elements of 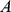 define a connection between dipolar sources in the immediate spatial neighborhood.

Let us define 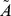, the discrete Laplacian over the geodesic surface at the first spatial neighborhood order as:
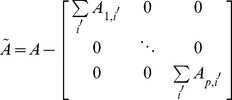
(2)


Note that the non-null entries of 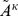 represent spatial connections between dipolar sources within the 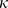 ^th^ order spatial neighborhood. We used the spatial smoothness model *W* introduced in Friston et al. (2008) [27], which is defined by:
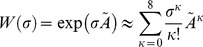
(3)where 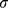 is a parameter that tunes the strength of spatial smoothness, varying between 0 and 1. In equation (3), the upper bound of summation was set to 8 as in Friston et al. (2008) [27]. Note that from equation (3), the spatial smoothness matrix *W* can be interpreted as a generalization of a discrete Laplacian over a large neighborhood order.

### Regularization Techniques

#### Maximum Entropy on the Mean (MEM) framework

In the MEM framework, we consider the amplitude of the sources *J* to be estimated as a multivariate random variable of dimension p, with a probability distribution 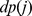. The MEM principle aims at estimating the distribution 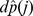 that provides “maximum uncertainty about missing information carried by the data” [39], with respect to some reference model assumed on *J* [24]. Regularization in this framework is introduced by writing the solution in the form of 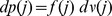, where the reference distribution *dν* expresses some assumptions on *J* and 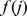 is a 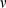
- density to be found such that it explains the data in average:
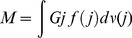
(4)


Among all the distributions *dp* satisfying the above constraint, the MEM solution 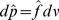 is the one with maximum ν-entropy [16], [24]. An interesting property of the MEM approach relies in its inherent flexibility for introducing constraints through the definition of the reference distribution *dν*. In this study, *dν* was defined using the parcellization model P(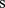) assuming brain activity to be described by K cortical parcels showing homogeneous activation state.

Each cortical parcel k is characterized by an activation state 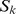, describing if the parcel is active 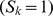 or not 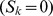. Assuming a collection of mutually independent parcels, the global *dν* was defined as a factorization of the joint probability distribution of the K parcels:
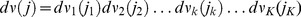
(5)with the following mixture model for each parcel:
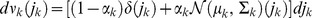
(6)where 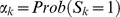 is the probability of the 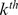 parcel to be active. Multivariate 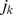 denotes the intensities of the 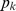 sources in the 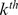 parcel. δ refers to the Dirac distribution allowing to “shut down” inactive parcels when 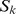 = 0. 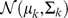 is a Gaussian distribution describing the intensities of the 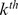 parcel when active (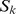 = 1); where 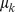 and 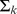 represents respectively the mean and the covariance of the 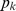 sources within the 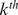 parcel. These parameters will be described in the next section.

The purpose of the present study was to evaluate different initialization of *dν* in the MEM framework, considering spatial modeling introduced using P(s) and local spatial smoothness within parcels. Once *dν* is initialized, the MEM solution 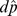 is obtained through the optimization of a convex function in a q-dimensional space (see Appendix S3 for details). Note that whereas MEM estimation was done iteratively at each time sample, the same clustering model P(s) was used over the whole time window of signal to localize.

#### Source localization methods within the MEM framework

When incorporating the parcels P(s) through *dν* in the MEM framework, the first step consists in the definition of the parameters *(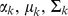)* of 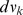 for each parcel (equation 6).

- A spatio-temporal Activation Probability Map (stAPM) was generated (see Appendix S4), by mapping the MSP coefficients of 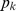 sources in the 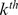 parcel (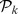) along time. Therefore, the probability of activation of the parcel 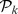 was initialized at each time sample 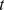 as 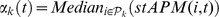.
- In the reference model (equation 6), we assumed the Gaussian distribution of the active state to be a zero mean distribution; therefore, *μ_k_* of each parcel was initialized to zero.
- The covariance matrix for each parcel is a time varying matrix Σ*_k_* (*t*) defined as follows:
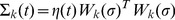
(7)where 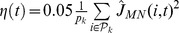 is a scaling factor for the covariance of each parcel 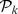, estimated using the average of the square of the mean activity provided by the Minimum Norm solution 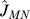 [22] within the 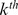 parcel. This scaling factor was arbitrarily initialized as 5% of the energy within each parcel. In equation 7, 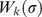 is defined as the 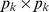 matrix controlling local spatial coherence within the parcel, obtained by selecting the rows and columns of 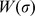 corresponding to the 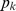 sources of the 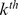 parcel.

Accordingly, under these assumptions, we propose the two following methods:

- *Maximum Entropy on the Mean at a specific cluster scale s (MEM-s)* consists in setting 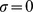 leading to 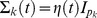, where 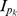 is a 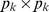 identity matrix.
- *Coherent–MEM-s at a specific cluster scale s (CMEM-s)* consists in setting 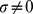 leading to 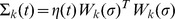. We have used 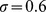, as suggested in Friston et al. (2008) [27], to introduce local spatial smoothness in each parcel.

For MEM-s and CMEM-s, we defined the reference distribution with mean *μ_k_*  = 0 with the hypothesis that we do not add much information a priori, since MEM provides inference on the mean of the distribution. On the other hand, we hypothesized that the initialization of the covariance matrix Σ*_k_* (*t*) for each parcel as 5% of the averaged energy of the Minimum Norm solution 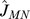 will ensure a proper scale for the intensity of the reference distribution.

#### Hierarchical Bayesian (HB) framework

Solving the MEG inverse problem within the HB framework offers the advantage of accommodating multiple priors and proposes inference techniques to select the most likely combination of priors using model selection approaches [26], [27], [40]–[44]. We thus chose HB as a key framework in which we could consider similar priors as the ones proposed for MEM thereby allowing an ideal comparison of the two approaches.

HB model allows integrating uncertainties at different levels, modeling the covariance in each level as linear combination of covariance components. The different levels are the sensor noise level and the source noise level.

At the sensor level (1^st^ level), the relationship between the MEG measurements (M) and the source amplitudes (*J*) is given by:
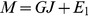
(8)


At the source level (2^nd^ level), the prior distribution of the source amplitudes (*J*) is given by:
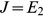
(9)where 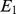 and 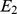 represents additive random fluctuations in the sensor and source space respectively. The a priori distribution of these additive random noises is a zero mean Gaussian distribution with spatial covariances 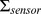 and 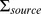 and temporal covariance *θ_t_*, such as:



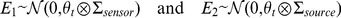
(10)Here θ_t_ was modeled as the identity matrix. The sensor spatial covariance 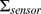 was modeled as:
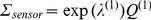
(11)Where 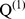 refers to a spatial covariance component, i.e., the identity matrix here, and 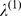 represents the corresponding hyper-parameter.

The source spatial covariance 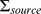 was modeled as a linear combination of the form:
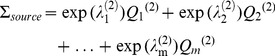
(12)where 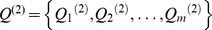 describes the spatial covariance components of the source level and 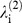 denotes the corresponding hyper-parameters, 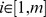. The exponential term on 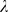 ensures the covariance model to be positive [27]. The hyper-parameters were estimated using Restricted Maximum Likelihood (ReML) algorithm, selecting the most relevant linear combination of covariance components (see details in Friston et al. (2002) [42]).

#### Source localization methods within the HB framework

In addition to two standard source reconstruction methods (IID and COH) implemented in SPM8 software package [27], we proposed a new method within this HB framework (COH-s).

*(a) COH-s: Coherent at a specific cluster scale s:* COH-s incorporates *spatially smooth extended parcels* within the HB framework, thus accounting for the same spatial priors as the ones considered in CMEM-s within the MEM framework. Three types of covariance components were considered: 1) Minimum norm component encoding independent sources 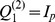, 2) global spatial smoothness 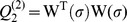 and 3) *K* locally spatially coherent parcels of 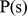 as independent covariance components denoted by (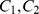
…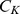). 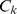 is a 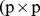) block matrix generated using the elements of 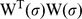, the block being extracted from the 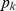 row and column indices of the 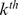 parcel, and zero elsewhere. 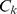 thus assumes local spatial smoothness over the whole 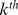 parcel. To summarize, COH-s assumes the following spatial covariance model:



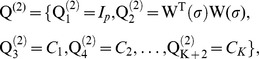
where *K* represents the total number of parcels at a specific spatial clustering scale 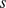.

*(b) Independent and Identically Distributed model (IID):* This model uses a single source covariance component encoding identically and independently distributed sources 
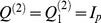
 (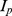 being a *p* dimension identity matrix). This method provides a minimum energy solution, similar to the one originally proposed by Hamalainen and IImoneimi (1994) [22].

*(c) Spatially Coherent Sources (COH):* This method provides a solution that is spatially smooth, similar to LORETA [23]. It consists in a model with two spatial components modeling respectively independent and spatially coherent sources:



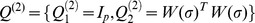



Note that COH-s is an extension of COH method using the concept of multiple parcels introduced in the Multiple Sparse Prior method proposed by Friston et al. (2008) [27]. Both COH-s and Multiple Sparse Prior methods are using several regional spatial covariance components. Whereas Multiple Sparse Prior models brain activity as small patches of coherent activity sparsely placed in the left and right hemispheres with a priori maximum variance at the center of the patch, COH-s incorporates *spatially smooth extended parcels.* In COH-s model, the non-zero terms of the diagonal of 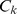 have a priori the same energy. Multiple Sparse Prior method was designed to localize focal “sparse” sources, and was proved to be efficient in cognitive studies [45]. As our objective was to localize spatially extended sources of epileptic activity, we proposed COH-s as a method in the HB framework to be compared with MEM-s and CMEM-s.

### Evaluation Using Realistic Simulations

We evaluated the performance of the five above-mentioned source localization methods in their ability to localize spatial extended sources. To perform this validation, we proposed a fully controlled environment to generate realistic simulations of MEG data mimicking the generators of epileptic spikes with different spatial extents, similarly to the evaluation proposed for EEG source localization in Grova et al. (2006) [16]. This section describes the validation dataset and validation metric used for the evaluation.

#### Ethics statement

Realistic simulations were generated using MEG data obtained from a patient with focal epilepsy showing normal tracing with no epileptic activity. This patient participated as a research subject of the project entitled: “Application of magnetoencephalography in the assessment of the epileptic focus” (Dr. E. Kobayashi being the principal investigator for this project). Written informed consent for this study was obtained from the subject as approved by the Research Ethics Committee of the Montreal Neurological Institute and Hospital (MNI/H). At its full board meeting of June 14, 2011, the Research Ethics Board (REB) of the MNI/H has endorsed the review of this project and found this research to be acceptable for continuation at the McGill University Healthcare Centers. The REB of the MNI/H acts in conformity with standards set forth in the (US) Code of Federal Regulations governing human subjects’ research and functioning in a manner consistent with internationally accepted principles of good clinical practice.

#### Validation dataset

The subject we selected to generate our realistic simulations had normal cortical surface segmented from his anatomical Magnetic Resonance Imaging data. This acquisition was done at the MEG center of Université de Montréal on a 275 channels CTF whole-head MEG system. The detection coils used in the system were first order radial gradiometers. The CTF system is equipped with reference sensors using a 3^rd^ order gradient correction to subtract background interferences. During the acquisition, the head position of the subject was tracked using localization coils placed on three fiducial points (nasion, left and right peri-auricular points).

A high resolution T1 weighted MRI was acquired on the same subject at the MRI center of the Montreal Neurological Institute. Co-registration between MEG sensors position and the anatomical T1-weighted MRI of the subject was obtained in three steps: (i) manual identification of the three fiducial points on the MRI, (ii) digitalization of the position of the fiducials on the head of the subject using a 3D Polhemus localizer and (iii) the rigid geometrical transformation between the MRI’s space and the subject’s space was obtained by fitting these points using Procrustes method [46].

A realistic head model was obtained by segmenting the surface of the brain from the subject’s anatomical T1-weighted MRI [47]. The distributed source model was obtained by segmenting the white/gray matter interface from the MRI using Brainvisa software (BrainVISA: http://www.brainvisa.info). The source model consisted in a realistic 3D mesh of the cortical surface (4203 vertices, 7 mm mesh). The forward matrix G (in equation 1) was computed using the Boundary Element Method (BEM) proposed by Kybic et al. (2006) [48]. A 1-layer BEM model consisting of only the inner skull surface was considered and estimated using OpenMEEG software (OpenMEEG: http://www-sop.inria.fr/odyssee/software/OpenMEEG/).

##### Simulation parameters

100 simulation configurations involving one extended source were generated. The position of each source was selected by choosing a seed point randomly on the cortical surface mesh. The spatial extent of each source was obtained by region growing around the seed following the cortical surface using different spatial neighborhood orders ranging from a source spatial extent *s_e_* = 2 (*∼*3 cm^2^) to *s_e_* = 6 (*∼*30 cm^2^). The amplitude of each vertex of the simulated source was set to 9.5 nA.m, generating an overall maximum signal of 1.5 pT for MEG when all the sources of the cortex were set active. This value has been chosen to mimic realistic amplitude of a typical epileptic spike.

The time course of the simulated sources was the time course of an epileptic spike modeled with three Gamma functions, although only signal around the main peak of the spike was analyzed (about 21 samples around the peak with a sampling rate of 600 Hz). Let us refer to 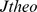 as the simulated theoretical current distribution obtained from the spatial distribution of the simulated sources together with the corresponding time course. Noise-free MEG data were then simulated by applying the forward model *G* to the simulated current density 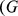 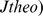. Realistic physiological noise was extracted from a three minutes segment of MEG background activity acquired on a patient with focal epilepsy showing normal traces without any epileptic discharge. MEG data acquired at 600 Hz were filtered between 0.3 Hz and 70 Hz. Periods with motion and eye blinks were excluded. Each noise-free simulated MEG signals were then corrupted by adding some real MEG background activity. In order to mimic MEG spikes averaging, 128 trials of 700 ms of MEG background activity were manually identified. For each simulation, 20 trials were randomly selected among the 128 trials, averaged and added to the simulated signal. The amplitude of all 128 trials was scaled to ensure a signal-to-background ratio of 1 (0 dB) for most superficial sources when using reference source amplitude of 9.5 nA.m along a patch of 6 cm^2^. Consequently, the simulation of deep sources resulted in simulated signals with lower amplitude than the superficial sources. Therefore, the Signal-to-Noise Ratio (SNR, defined as the ratio of maximum activity at the peak to the standard deviation of the background activity) of the realistic simulated data varied depending upon the location and extent of the underlying sources.

In order to investigate the influence of the spatial clustering scale *s* of P(s) for MEM-s, CMEM-s and COH-s, we tested the performance of the methods when varying the spatial clustering scale *s* from *s* = 3 (K∼200 parcels) to *s* = 6 (K∼40 parcels), for each source spatial extent varying from *s_e_* = 2 (∼3 cm^2^) to *s_e_* = 6 (∼30 cm^2^), and for each of the 100 random source positions, leading to a total of 4(*s*)×5(*s_e_*)×100(configurations)×5 methods = 10,000 source localizations.

We also performed the following investigations: 1) to compare the performance of the methods that uses DDP model (MEM-s, CMEM-s and COH-s) when initializing parcels P(s) either with the data of interest or with some background MEG activity and 2) to compare the ability of the methods to localize single spike versus averaged spike data (average of 20 spikes). For these two tests we considered 50 source configurations of spatial extent *s_e_* = 3 (∼7 cm^2^) and the methods involving P(s) were localized using a spatial clustering scale of *s* = 5.

All the simulations were performed with Matlab (R2010a) using the simulation environment Pipeline System for Octave and Matlab (PSOM) [49].

#### Validation metric

In this section we describe the validation metrics used to evaluate the detection accuracy of the source localization methods presented in ***section: Regularization techniques***. Note that the solution of the inverse problems was estimated and evaluated at one single time sample, at the peak of the spike.

*(a) Area Under the ROC Curve (AUC):* To assess the detection ability of the different localization methods, we used the Area Under the Receiver Operating Characteristic (ROC) curve [50], denoted by AUC, as a detection accuracy index assessing the sensitivity to the spatial extent of the sources. This metric was adapted by Grova et al. [16] to fit the context of a distributed source model. We chose the AUC index as the validation metric mainly because of the difficulty of providing a valid statistical threshold for all the proposed methods. The AUC index was estimated at the main peak 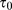 of the simulated spike. We estimated the energy 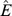 of the current density distribution at 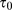, for each localization. To compare 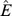 with 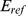
(energy of the simulated current density distribution), 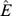 and 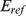 were first normalized between 0 and 1 for each dipole *i*: 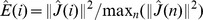 and 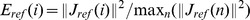. We quantified the specificity and sensitivity of the localization method by varying a threshold *β* between 0 and 1 and considering a dipole *i* to be active if 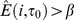. ROC curves were then obtained by plotting sensitivity (*β*) against (1– specificity(*β*)). AUC was finally estimated to assess detection accuracy. In our study, a value of AUC>0.8 was considered to be good detection accuracy, suggesting 80% of detections were accurate.

However, to interpret the area under the ROC curve as a detection accuracy index, one should provide the same number of active and inactive sources to the ROC analysis [16]. Indeed, in the context of distributed source evaluation, the estimation of AUC is biased by the fact that among the 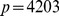 dipoles of the source model, only few dipoles (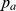) were actually active compared to the large number of inactive dipoles 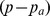. A more accurate estimation of AUC was obtained by using as many inactive sources as active sources during the evaluation. This was done by randomly selecting 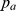 inactive or fictive sources among the 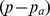 available either within the immediate spatial neighborhood of the simulated sources (AUC_close_) or within far local maxima of the source localization results (AUC_far_). The final AUC index was computed as the mean of AUC_close_ and AUC_far_, thus providing a metric assessing both the ability of the method to focalize the reconstructed activity and the eventual generation of spurious sources far from the simulated one (see [16] for more details).

*(b) Mean Square Error (MSE):* To assess the ability of the methods to accurately recover the amplitude of the simulated current density (*Jtheo*), we estimated the mean square error (MSE) [16] between the simulated current amplitude and the reconstructed one over the whole brain at the peak of the spike. Lower MSE values indicate that the method is able to recover the current amplitude with high accuracy.

*(c) Minimal geodesic distance to the source (Dmin):* To quantify source localization accuracy, we estimated the minimum geodesic distance between the dipolar source showing the global maximum of reconstructed activity source and the closest dipole belonging to the simulated source. This geodesic distance following the circumvolutions of the cortical surface was denoted by Dmin [16]. Solutions for which this global maximum was localized on the wrong hemisphere, Dmin could not be estimated since the surfaces of the two hemispheres were not connected geodesically. Therefore, Dmin was finally set at the largest Dmin value obtained over all source configurations in such cases. A value of Dmin close to 0 indicated that the maximum of reconstructed activity source was found within the simulated source.

In addition, AUC was measured as a function of eccentricity to check for the influence of the depth of the source on detection accuracy. The eccentricity of a simulated source was defined as the distance between the seed point of the spatially extended source to the center of the head, whereby the deepest source have a lower eccentricity value (10 mm) and the most superficial ones have a higher eccentricity value (90 mm). Sources with eccentricity ranging between 40 mm and 60 mm corresponded mainly to mesio-temporal sources and the ones with eccentricity less than 40 mm corresponded to the sub-cortical sources.

## Results

### Qualitative Assessment

The purpose of this first section is to evaluate qualitatively the performance of three simulations together with the corresponding validation metrics AUC, MSE and Dmin. To visualize the results, we showed the absolute value of the reconstructed activity at the peak of the simulated spike, thresholded upon the level of background activity [51].

**Figure 2:**
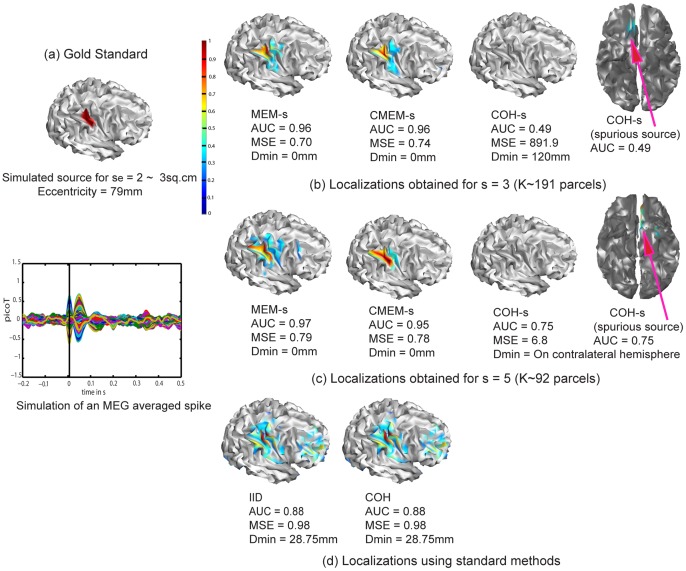
Qualitative assessment. Visual analysis of source localization results together with Area Under the ROC curve (AUC) values for a simulated source of spatial extent *s_e_* = 2 and eccentricity 79 mm. All source localization results are presented as the absolute value of the current density at the peak of the spike, normalized to its maximum activity and thresholded upon the level of background activity [51]. (a) Theoretical simulated source: spatial extent of the cortical source and associated simulated MEG signal for all MEG sensors (data being localized within a window of 20 time samples around the first peak of the spike). (b) Source localization results obtained for MEM-s, CMEM-s and COH-s at *s* = 3. (c) Source localization results obtained for MEM-s, CMEM-s and COH-s at *s* = 5. (d) Source localization results obtained for IID and COH.

Figure 2 illustrates the ability of the five evaluated methods to localize a right occipito-parietal source with an extent of *s_e_* = 2(∼3 cm^2^) and an eccentricity of 79 mm (superficial source). Note the AUC values were in agreement with visual inspection. We observed that methods MEM-s and CMEM-s were the most accurate in detecting the spatial extent of the source (AUC>0.90, MSE ≈ 0.70 and Dmin = 0 mm for MEM-s and CMEM-s at *s* = 3 and 5). IID and COH showed slightly less accurate localization (AUC = 0.88, MSE = 0.98, Dmin = 28.7 mm), probably due to the presence of low amplitude frontal spurious sources. Note that both IID and COH underestimated the spatial extent of the source equally and exhibited very similar solutions. For COH method, ReML model selection actually pushed forward the minimum energy prior over the spatial smoothness prior (cf. ReML estimates for COH, 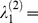 0.057 and 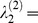 0 in equation (12)). This makes COH interesting when localizing focal sources, as it is able to choose between the minimum energy solution for more focal sources and the spatial smoothness solution for spatially extended sources. Finally, in this specific case, COH-s failed to find the simulated occipito-parietal source (Figure 2b, 2c), as it exhibited a spurious source (Dmin = 120.3 mm at *s* = 3 and at *s* = 5 the maximum activity was found on wrong hemisphere) in the deep fronto-mesial region, resulting in poor localization accuracy (AUC = 0.49 and MSE = 891 at *s* = 3 and AUC = 0.75 and MSE = 6.8 at *s* = 5).

We also illustrated the impact of the clustering scale *s* in P(s) (see Figure 2b and Figure 2c). MEM-s and CMEM-s provided similar AUC values at *s* = 3 and *s* = 5. CMEM-s reproduced accurately the extent of the source following a smooth diffusion along the cortical surface (AUC = 0.96 and MSE = 0.74 at *s* = 3, AUC = 0.95 and MSE = 0.78 at *s* = 5), whereas MEM-s (AUC = 0.96 and MSE = 0.70 at *s* = 3, AUC = 0.97 and MSE = 0.79 at *s* = 5) recovered a similar spatial extent with less local smoothness. The profile of reconstructed intensities using MEM-s is actually similar to typical profiles observed in a minimum norm solution; however, it exhibits a larger contrast over the actual extent of the source. We also noticed a slight over-estimation of the extent of the source when using MEM-s and CMEM-s at *s* = 5. On the other hand, increasing the clustering scale *s* from 3 to 5 had very little impact on the accuracy of COH-s localization in this example, probably because of the presence of spurious sources. Whereas COH-s did show a larger AUC value of 0.75 for *s* = 5 when compared to AUC = 0.49 at *s* = 3, an accurate low intensity source was found at *s* = 5, but did not pass Otsu’s threshold [51], as the intensity of the spurious source was larger.

**Figure 3:**
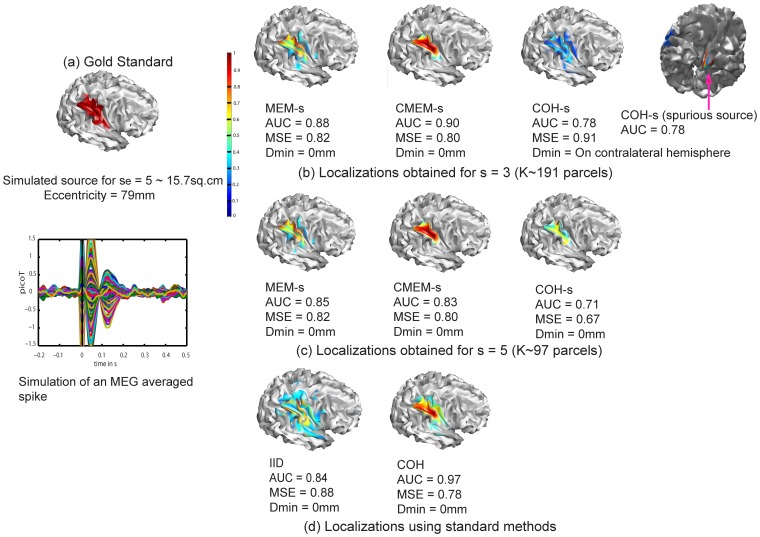
Qualitative assessment. Visual analysis of source localization results together with Area Under the ROC curve (AUC) values for a simulated source of spatial extent *s_e_* = 5 and eccentricity 79 mm. Remaining information same as in Figure 2.

Figure 3 illustrates the ability of the five methods to localize the same right occipito-parietal source but more spatially extended (extent *s_e_* = 5 (∼15.7 cm^2^), eccentricity = 79 mm). All methods were able to localize this source, but the spatial extent of the source has been slightly under-estimated. COH was the most accurate in reproducing the source spatial extent with AUC = 0.97, MSE = 0.78 and Dmin = 0 mm. In this example, COH favored the spatial smoothness solution (cf. ReML estimates for COH, 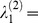 0 and 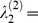 0.28), hence, provided better localization than for the previous example. IID showed less accurate localizations (AUC = 0.84, MSE = 0.88) due to under-estimation of the spatial extent of the simulated source, whereas the maximum of activity was accurately localized (Dmin = 0 mm, Figure 3d). MEM-s and CMEM-s reproduced the source spatial extent with good accuracy at *s* = 3 and *s* = 5 (AUC = 0.88 and 0.85 for MEM-s, 0.90 and 0.83 for CMEM-s, with MSE∼0.80 and Dmin = 0 mm). CMEM-s was able to detect the local maximum activity of the source following a smooth diffusion along the cortical surface; however, the source spatial extent was slightly under-estimated. MEM-s was able to localize the source but lacked smooth diffusion along the cortical surface. COH-s provided less accurate source localization for both *s* = 3 and 5 (AUC = 0.78, and 0.71). At *s* = 3 COH-s was able to detect the source and its spatial extent but also presented a higher intensity deep spurious source (cf. Dmin: maximum located in the wrong hemisphere, MSE = 0.91) (Figure 3b). At *s* = 5, COH-s detected the source with a low intensity and under-estimated its spatial extent (AUC = 0.71), whereas the maximum was accurately localized (MSE = 0.67, Dmin = 0 mm). Note that the size of the parcels used in COH-s (*s* = 3 and 5) are smaller than the source spatial extent.

**Figure 4:**
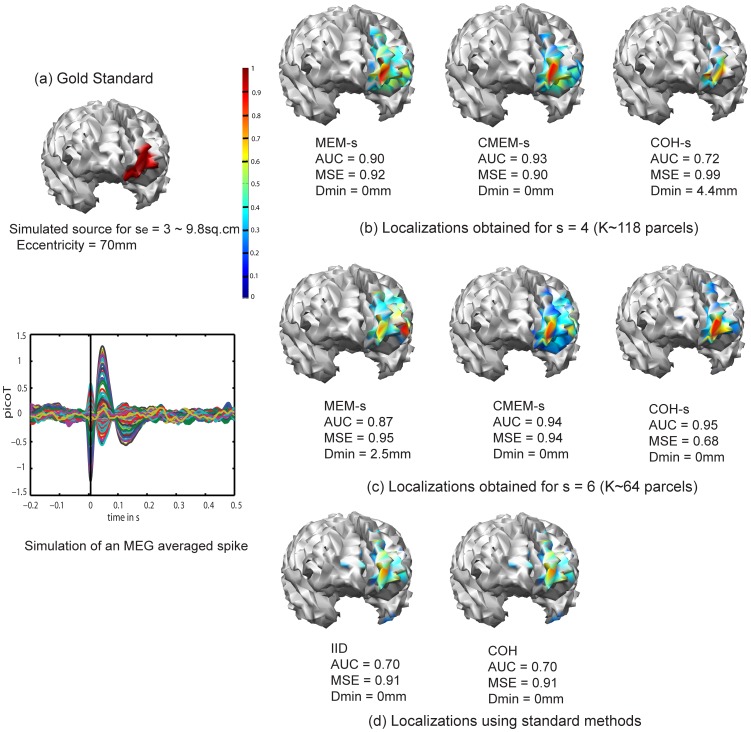
Qualitative assessment. Visual analysis of source localization results together with Area Under the ROC curve (AUC) values for a simulated source of spatial extent *s_e_* = 3 and eccentricity 70 mm. Remaining information same as in Figure 2, except that cluster scales *s* = 4 (b) and *s* = 6 (c) were considered for MEM-s, CMEM-s and COH-s.

Figure 4 illustrates the ability of the five methods to localize a deeper and less extended left orbito-frontal mesial source (spatial extent *s_e_* = 3 (∼9.8 cm^2^), eccentricity = 70 mm). Overall the deeper aspects of the source were difficult to localize. MEM-s and CMEM-s provided good localization of some superficial aspects of the source (MEM-s with AUC = 0.90, MSE = 0.92 and Dmin = 0 mm at *s* = 4 and AUC = 0.87, MSE = 0.95 and Dmin = 2.5 mm at *s* = 6, CMEM-s with AUC = 0.93, MSE = 0.90 and Dmin = 0 mm at *s* = 4 and AUC = 0.94, MSE = 0.94 and Dmin = 0 mm at *s* = 6). Increasing the clustering scale *s* from 4 to 6 had no impact on the localization of MEM-s and CMEM-s (Figure 4b and 4c). On the other hand, increasing the clustering scale *s* from 4 to 6 had a great impact on the accuracy of COH-s localization (AUC = 0.72, MSE = 0.99 and Dmin = 4.4 mm for *s* = 4 to AUC = 0.95, MSE = 0.68 and Dmin = 0 mm for *s* = 6). COH-s at *s* = 6 provided the most accurate localization of this source. COH and IID provided the least accurate localizations (AUC = 0.70, MSE = 0.91 and Dmin = 2.5 mm, Figure 4d). In this last case, COH actually chooses the minimum norm solution when localizing focal sources (cf. ReML estimates for COH, 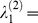 0.024 and 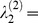 0).

### Effect of the Spatial Extent of the Simulated Sources

Table 1 reports the medians of AUC values obtained over 100 source configurations for all the five methods and all the source spatial extents *s_e_* = 2, 3, 4, 5, and 6. For this comparison, MEM-s, CMEM-s and COH-s were considered using a clustering scale of *s* = 5. For all the spatial extents, COH, MEM-s and CMEM-s exhibited median AUC values greater than 0.8, indicating overall good detection accuracy with these methods. COH-s showed median AUC values greater than 0.8 for all extents less than *s_e_* = 5. Note that for *s_e_* ≥5, COH-s provided low median AUC values (<0.8) when localizing using a clustering scale *s* = 5. IID showed median AUC values lower than 0.8 for all the extents.

**Table 1:** AUC medians obtained over 100 source configurations for all five methods and all five spatial extents *s_e_*.

	Methods - AUC median				
Spatial Extents	IID	COH	MEM-s (s = 5)	CMEM-s (s = 5)	COH-s (s = 5)
*s_e_* ** = 2**	0.77	**0.80**	**0.86**	**0.89**	**0.82**
*s_e_* ** = 3**	0.78	**0.82**	**0.88**	**0.89**	**0.86**
*s_e_* ** = 4**	0.75	**0.85**	**0.87**	**0.89**	**0.83**
*s_e_* = **5**	0.74	**0.85**	**0.86**	**0.86**	0.77
*s_e_* ** = 6**	0.74	**0.87**	**0.83**	**0.84**	0.72

(in bold font: median AUC>0.80).

**Figure 5:**
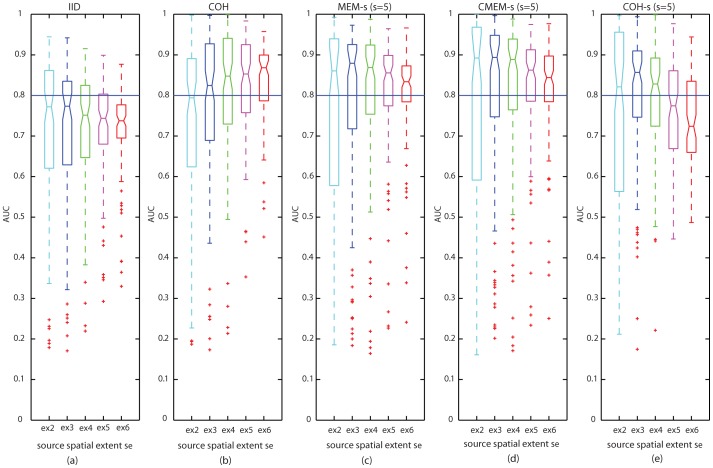
Effect of source spatial extent *s_e_*. Distribution of AUC results using boxplot representations over 100 simulations of randomly placed sources for all source localization methods. x-axis from left to right: source spatial extent *s_e_* = 2 (cyan), *s_e_* = 3 (blue), *s_e_* = 4 (green), *s_e_* = 5 (purple) and *s_e_* = 6 (red); (y-axis: AUC values); (Horizontal line, AUC = 0.8). MEM-s, CMEM-s and COH-s results are reported when using a spatial clustering scale *s* = 5. (a) IID, (b) COH, (c) MEM-s, (d) CMEM-s and (e) COH-s.

The above results are presented with more details in Figure 5, which illustrates the distributions of AUC values for all the five methods using boxplot representations for *s_e_* = 2, 3, 4, 5 and 6. For this boxplot representation, MEM-s, CMEM-s and COH-s are considered using a clustering scale *s* = 5. We observed an overall very good accuracy for MEM-s and CMEM-s source localizations (median AUC>0.8 for all spatial extents). However, we noticed that their accuracy in localization decreased slightly when increasing the source spatial extent (Figure 5c and 5d), but still remained among the most accurate methods. For COH, we observed that accuracy in the localization of sources showed slight tendency to increase with the increase in source spatial extent (Figure 5b). For COH-s at *s* = 5, we observed poor localization accuracy for *s_e_* = 5 and 6, and excellent accuracy for smaller sources *s_e_*<5 (Figure 5e). Overall IID was not really sensitive to the spatial extent of the sources, showing the smallest AUC values (Figure 5a).

Table 2 reports the median and L1 dispersion (i.e., the average of the absolute deviations from the median) of MSE and Dmin metrics obtained over 100 source configurations for the five methods and all the source spatial extents *s_e_* = 2, 3, 4, 5, and 6. For all the spatial extents, MEM-s and CMEM-s provided very similar MSE values (∼0.90) indicating an accurate recovery of the source amplitude for all the spatial extents. Similar MSE values were found for COH and IID for all the spatial extents. From all the five methods, only COH-s showed the highest MSE error with a large standard deviation. Overall, MSE was not very informative to compare localization methods in our context, except for detecting important false detection when using COH-s.

**Table 2:** Median (Med) and L1 Dispersion (Disp) of MSE and Dmin over 100 source configurations for all five methods, all five spatial extents *s_e_* = *2,3,4,5,6* and all four clustering scale *s = 3,4,5,6* in MEM-s, CMEM-s and COH-s.

Methods		Metrics	s_e_ = 2 Med(Disp)	s_e_ = 3 Med(Disp)	s_e_ = 4 Med(Disp)	s_e_ = 5 Med(Disp)	s_e_ = 6 Med(Disp)
**IID**		*MSE Dmin(mm)*	1.1 (0.14) 4.9(45.0)	0.93(0.08) 0(21.8)	0.90(0.05) 0(17.9)	0.91(0.04) 0(9.1)	0.92(0.02) 0(5.4)
**COH**		*MSE Dmin(mm)*	1.1(0.16) 0(44.8)	0.91(0.12) 0(19.5)	0.86(0.11) 0(14.0)	0.82(0.07) 0(6.9)	0.83(0.05) 0(5.1)
**MEM-s**	***s = 3***	*MSE Dmin(mm)*	0.94(0.08) 9.2(28.7)	0.91(0.08) 0(20.3)	0.90(0.07) 0(13.3)	0.91(0.05) 0(5.4)	0.93(0.03) 0(3.6)
	***s = 4***	*MSE Dmin(mm)*	0.94(0.07) 11.7(29.1)	0.92(0.07) 2.6(16.1)	0.91(0.06) 0(13.6)	0.92(0.05) 0(5.5)	0.93(0.03) 0(4.8)
	***s = 5***	*MSE Dmin(mm)*	0.96(0.06) 12.3(28.7)	0.92(0.07) 4.2(18.1)	0.92(0.06) 0(14.0)	0.93(0.04) 0(6.7)	0.93(0.03) 0(3.5)
	***s = 6***	*MSE Dmin(mm)*	0.96(0.05) 17.9(31.7)	0.93(0.06) 4.7(22.1)	0.92(0.05) 0(14.3)	0.93(0.04) 0(7.7)	0.93(0.03) 0(4.5)
**CMEM-s**	***s = 3***	*MSE Dmin(mm)*	0.94(0.08) 10.7(34.2)	0.90(0.08) 0(18.7)	0.89(0.07) 0(8.9)	0.90(0.06) 0(5.7)	0.90(0.04) 0(3.9)
	***s = 4***	*MSE Dmin(mm)*	0.95(0.07) 12.9(31.7)	0.91(0.09) 0(19.5)	0.89(0.07) 0(9.9)	0.90(0.06) 0(6.7)	0.90(0.04) 0(3.2)
	***s = 5***	*MSE Dmin(mm)*	0.96(0.06) 12.0(26.7)	0.90(0.08) 3.5(20.7)	0.89(0.07) 0(11.0)	0.91(0.06) 0(6.8)	0.91(0.04) 0(4.1)
	***s = 6***	*MSE Dmin(mm)*	0.96(0.06) 14.3(35.6)	0.91(0.07) 0(21.1)	0.90(0.07) 0(12.0)	0.91(0.06) 0(5.4)	0.90(0.04) 0(3.1)
**COH-s**	***s = 3***	*MSE Dmin(mm)*	6.67(44.4) 59.5(50.9)	4.04(13.0) 28.7(35.7)	3.26(15.5) 25.4(34.5)	3.05(17.1) 25.6(29.3)	2.86(10.1) 29.5(31.5)
	***s = 4***	*MSE Dmin(mm)*	2.87(19.1) 41.6(56.7)	1.26(6.4) 4.6(41.9)	1.06(119) 0(29.0)	1.13(2.50) 0(17.8)	1.27(2.02) 0(13.9)
	***s = 5***	*MSE Dmin(mm)*	2.28(5.2) 27.2(55.7)	0.81(0.93) 0(28.4)	0.77(0.53) 0(19.0)	0.79(2.07) 0(21.1)	0.83(0.36) 0(14.6)
	***s = 6***	*MSE Dmin(mm)*	2.09(3.4) 22.7(55.4)	0.84(13361) 0(27.1)	0.70(1.5) 0(18.7)	0.77(0.44) 0(12.0)	0.78(0.31) 0(7.3)

MSE = Mean Squared Error, Dmin = Minimum geodesic distance between the local extrema of the reconstructed source and the simulated source, L1 Dispersion = the average of the absolute deviations from the median.

The minimum geodesic Dmin distance indicated that in most cases the maximum of reconstructed activity was located within the simulated sources (Dmin = 0), except for some large false detections observed with COH-s at smaller cluster scales *s* and for the more focal sources *s_e_* = 2. Keeping in mind that the average distance between two vertices of the cortical surface was 7 mm, Dmin results at *s_e_* = 2 illustrated that for MEM-s, CMEM-s and IID, whenever Dmin was not 0, the maximum activity was found within the 1^st^ or 2^nd^ spatial neighborhood order of the source, which is still quite close (Dmin∼10 mm).

Whereas Table 2 showed that, except for clear mis-localizations (cf. COH-s for *s* = 3), all methods performed similarly well according to standard localization metrics (MSE and Dmin), only AUC results (Tables 1 and Figure 5) were able to illustrate that MEM-based methods were indeed sensitive to the spatial extent of the sources. In other words, most methods localized accurately the maximum intensity of the sources, but only methods using a DDP model were sensitive to their spatial extents.

### Effect of the Clustering Scale *s*

**Figure 6:**
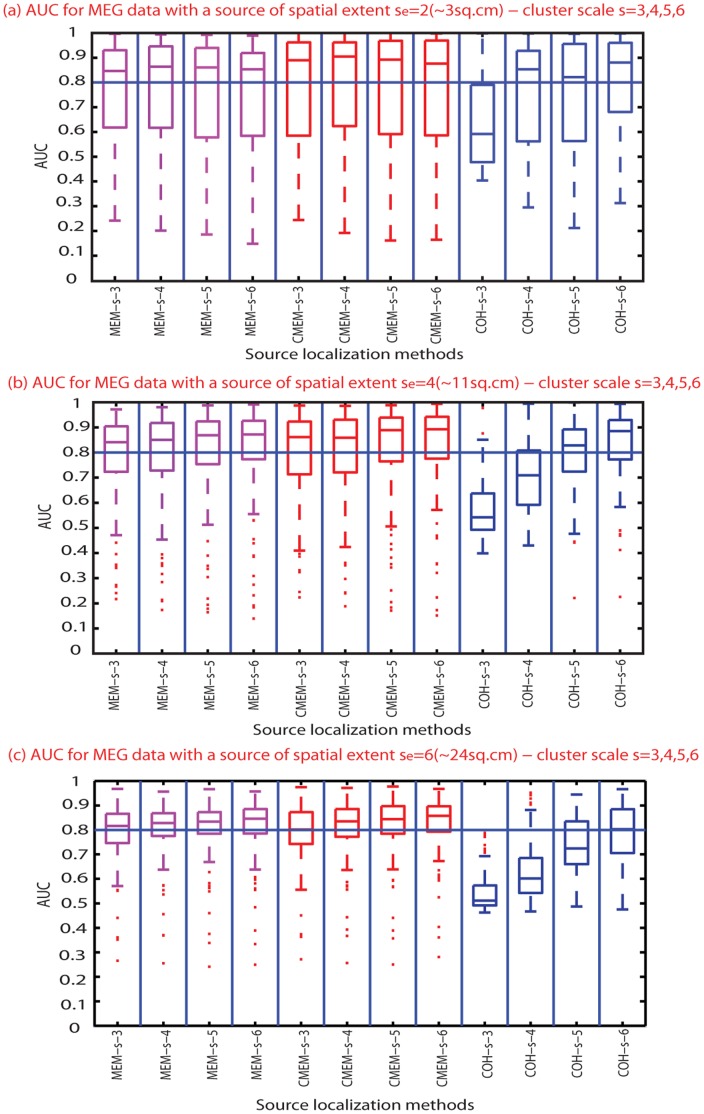
Effect of spatial clustering scale *s*. Distribution of AUC results using boxplot representations over 100 simulations of randomly placed sources for all source localization methods (x-axis from left to right: MEM-s with *s* from 3 to 6 (purple), CMEM-s with *s* from 3 to 6 (red), COH-s with *s* from 3 to 6 (blue), (y-axis: AUC values); (Horizontal line, AUC = 0.8). (a) Evaluation using simulated sources of spatial extent *s_e_* = 2 (∼3 cm^2^). (b) Evaluation using simulated sources of spatial extent *s_e_* = 4 (∼11 cm^2^). (c) Evaluation using simulated sources of spatial extent *s_e_* = 6 (∼24 cm^2^).

This section aims at assessing the impact of the clustering scale *s* in MEM-s, CMEM-s and COH-s methods that are all using the DDP model, P(s). Figure 6 shows the distribution of AUC values for these three methods, at the different clustering scales (*s* = 3, 4, 5, and 6) and for three spatial extents of the source (*s_e_* = 2, 4 and 6). Distributions of AUC values illustrated that the choice of the underlying clustering scale *s* had no impact on MEM-s and CMEM-s detection accuracy. On the other hand, accuracy of COH-s clearly increased when increasing clustering scale *s*. Figure 6, Figure 2b and Figure 3b demonstrate the poor performance of COH-s at *s* = 3. We observed that COH-s provided accurate localization for sources with spatial extent *s_e_* lower than the clustering scale *s*. However, this was not the case for the smallest spatial extent, *s_e_* = 2. In this case, even though the clustering scale *s* = 3 was greater than the spatial extent *s_e_* = 2, the overall detection accuracy for this spatial extent was accurate only when *s* >3 (Figure 2b and Figure 6).

Table 2 reports the median and L1 dispersion of MSE and Dmin values for different cluster scales for MEM-s, CMEM-s and COH-s. Similarly to AUC findings, we found that MSE and Dmin remained unaffected by the size of the clusters in MEM-s and CMEM-s. For COH-s we found that MSE and Dmin indicated accurate localization as soon as the cluster scale was larger than 3 (*s* >3), whereas performances at any scale *s* were very weak for focal sources at *s_e_* = 2.

### Effect of the Depth of the Simulated Sources

**Figure 7:**
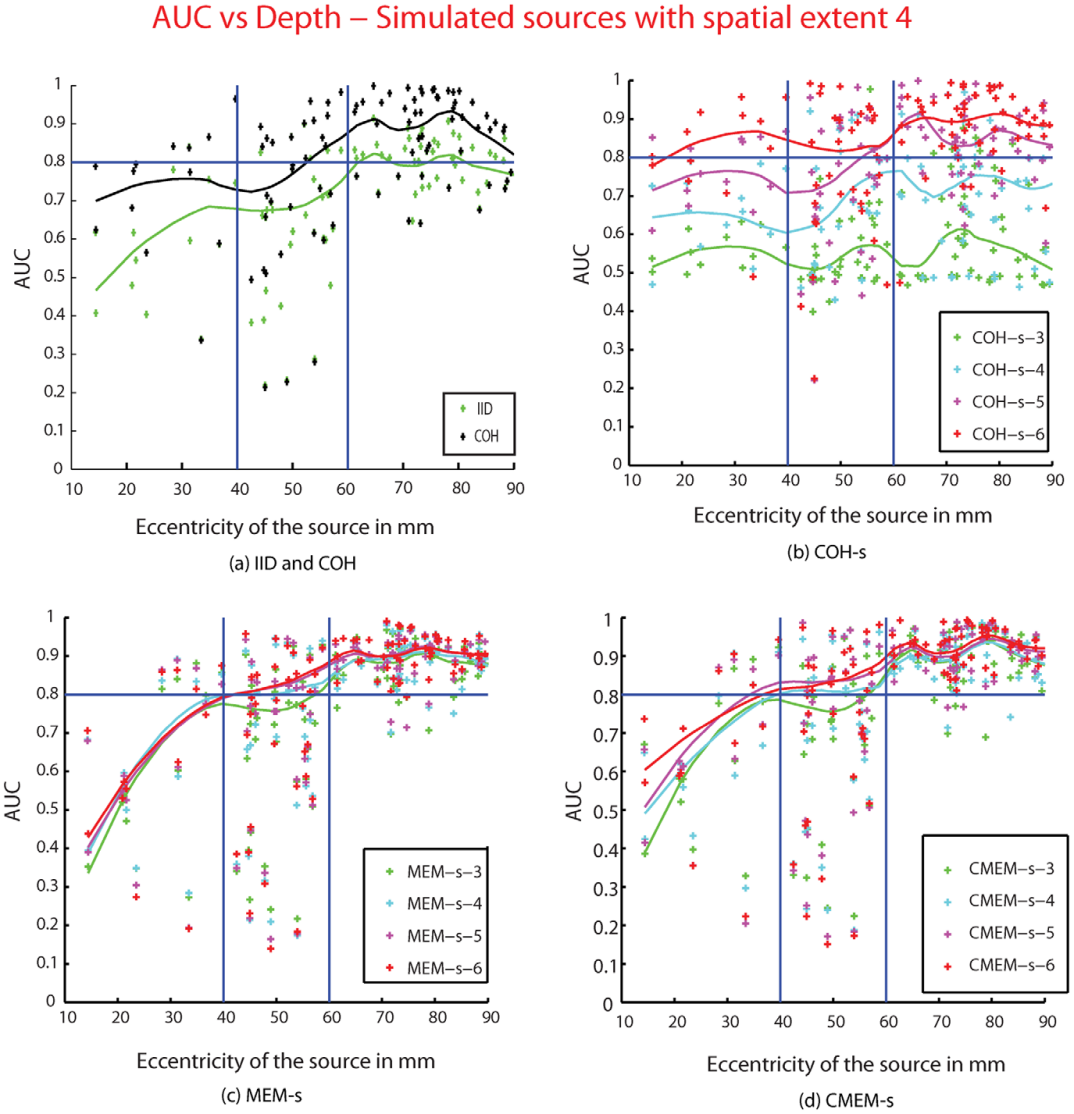
Effect of depth of the sources. Plot showing AUC values as a function of the eccentricity (in mm) of the 100 simulated sources (with spatial extent *s_e_* = 4). (a) IID (green), COH (black), (b) COH-s, (c) MEM-s, (d) CMEM-s. For the methods MEM-s, CMEM-s and COH-s, results for the different spatial clustering scale *s* are color coded as *s* = 3 (green), *s* = 4 (cyan), *s* = 5 (purple), and *s* = 6 (red). The solid lines are the moving average of the AUC values for the respective methods. Horizontal line, AUC = 0.8, Vertical lines: eccentricity = 40 mm and 60 mm.

We assessed the effect of the depth of sources on detection accuracy by plotting for each method, AUC as a function of eccentricity of the source for source spatial extents *s_e_ = *2,3,4,5 and 6. We illustrate the results of only one source spatial extent *s_e_* = 4 in Figure 7. The solid lines in Figure 7 correspond to the local moving average of the AUC values for each method. For all the source spatial extents *s_e_*, COH-s (for *s*>*s_e_*), MEM-s, CMEM-s and COH were able to localize most superficial sources (eccentricity >60 mm) with high accuracy (AUC >0.90). For sources with eccentricity ranging between 40 mm and 60 mm, which corresponded mainly to mesio-temporal sources, almost all the methods showed lower localization accuracy than for the superficial sources. Notably, COH-s at *s* = 6, MEM-s and CMEM-s still provided relatively good localization accuracy for these mesio-temporal sources (most AUC values >0.8). However, none of the methods (except COH-s at *s* = 6) were able to localize accurately deep sources (eccentricity<40 mm). COH-s at *s* = 6 performed better than other methods when localizing deep sources, although AUC was greater than 0.8 for *s_e_ = *4 only (Figure 7). IID exhibited poor localization accuracy for all the deep and mesio-temporal sources, although it provided better localization accuracy for superficial sources (AUC∼0.8). Figure 7b clearly demonstrated that when increasing the clustering scale *s*, COH-s showed an increase in detection accuracy for all sources; this was observed for the other source spatial extents too. From figure 7c and 7d, we observed that MEM-s and CMEM-s showed no notable influence of the clustering scale *s* on detection accuracy, which was observed for the other source spatial extents too.

This analysis also confirmed that most of the low AUC values considered as outliers in boxplot distributions, presented in Figure 5 and 6, were mainly due to the weak localization of deep sources.

### Effect of Parcellization using Data of Interest or Background Activity

**Figure 8:**
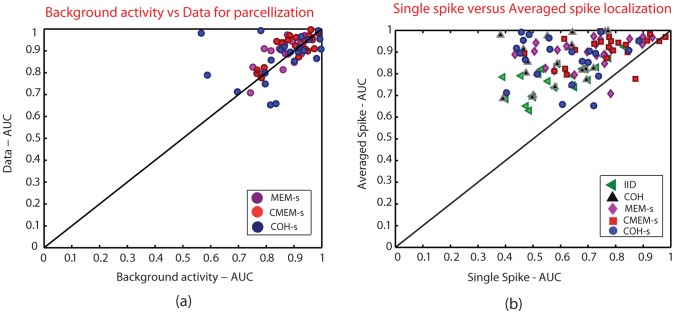
Comparison of detection accuracy AUC. Plot showing the (a) effect of background activity versus data of interest for the parcellization: MEM-s (purple), CMEM-s (red) and COH-s (blue) (x-axis: AUC value for Baseline, y-axis: AUC value for data), and (b) effect of single spike localization versus average spike localization: IID (green), COH (black), MEM-s (purple), CMEM-s (red) and COH-s (blue) (x-axis: AUC value for single spike localization, y-axis: AUC value for averaged spike localization).

Figure 8a illustrates the effect of initializing the parcellization P(s) (cf. Appendix S1) when using some MEG background activity or the data of interest, whereas only data of interest was considered to initialize the other parameters (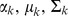). This investigation was performed using 50 source configurations with source spatial extent *s_e_* = 3 (∼7 cm^2^) and the methods involving P(s) were localized using a spatial clustering scale of *s* = 5. As localization of deeper sources would provide lower AUC values (***see section: Effect of the depth of the simulated sources***), in order not to bias our comparisons, we decided to consider only the most superficial sources with an eccentricity greater than 70 mm for this section i.e., 22 out of the 50 simulated sources were thus considered.

We can see that there was no impact on the detection accuracy of MEM-s, CMEM-s and COH-s when using the data of interest or MEG background activity to initialize the DDP model (Figure 8a), suggesting the importance of using a parcellization, but the accuracy of such a parcellization was not an issue.

### Effect of Single Spike versus Averaged Spike Localization

Figure 8b illustrates the comparison between detection accuracy of all the five methods when localizing single spike versus the average of 20 spikes. For this investigation, we used the same superficial source configurations as for the previous evaluation (***section: Effect of Parcellization using data of interest or background activity***). As expected, results showed a higher accuracy for the averaged data than the single spike data. We can also notice that the percentage of single spike simulations that were localized with a good detection accuracy (i.e., AUC>0.8) were respectively: CMEM-s = 37%, MEM-s = 18%, COH-s = 9%, COH = 4%, IID = 4%. These results suggest the robustness of both MEM-s and CMEM-s methods to low SNR conditions.

## Discussion

We have presented an evaluation of source localization methods for their ability to localize spatially extended sources of epileptic activity by incorporating realistic spatial models. We proposed three new source localization techniques that can detect the location of the sources with a good sensitivity to their spatial extent when using MEG data: MEM-s, CMEM-s and COH-s.

### Source Localization of Spatially Extended Sources

To be detectable from background activity on MEG, epileptic discharges need to be associated with spatially extended generators [11], [13], [14], [52], [53]. Several studies have been done on reconstructing extended cortical sources based on distributed model using EEG or MEG data [31], [36], [54]. Other studies have been proposed in the context of extended cortical patches and beamformer approaches; these methods are extensions or generalizations of dipole scanning approaches [11], [55]–[61]. All these methods indeed showed sensitivity for source spatial extents based on either controlled simulations [11], [55]–[58], [60], [61] or intracranial stereotactic EEG recordings [59]. However, our investigation involved a more detailed and larger range of spatial extents (3 cm^2^−30 cm^2^) than the ones evaluated in these studies (3 cm^2^−12 cm^2^ [11], 7 cm^2^−10 cm^2^ [56], 0.14 cm^2^−6 cm^2^ [57], 0.5 cm^2^−20 cm^2^ [58], and 0.09 cm^2^−2.2 cm^2^ [61]). In fact, the study by Birot et al. [58] simulated a range of spatially extended EEG sources very similar to ours and validated their localization methods using the validation metric (AUC) we proposed in Grova et al. (2006) [16]. Whereas, their proposed method, 4-EXO-MUSIC performed very well for large superficial sources (AUC>0.8 for 6 cm^2^−20 cm^2^), poor performances were observed for estimated sources smaller than 4 cm^2^ (AUC<0.6 for extent ≤4 cm^2^). On the other hand, our proposed MEM methods were providing very accurate localization (AUC>0.8) for all spatial extents considered in our study (3 cm^2^−30 cm^2^).

Standard localization metrics (Dmin and MSE) demonstrated overall good accuracy for most of the evaluated methods (except COH-s when *s<s_e_* and *s_e_* = 2), suggesting that the maximum of the activity was accurately localized in most cases. More importantly, only AUC metric was able to characterize better the ability of some methods to recover accurately the spatial extent of the sources, suggesting AUC to be a more appropriate metric in this context [16].

Whereas it is generally accepted that the minimum norm model (IID) is suitable for localizing the maximum of the activity [22], our results showed that IID was not sensitive to the spatial extent of the generators. Our findings are in agreement with the study of Ding (2009) [62], which demonstrated that a minimum norm model failed to recover the continuous cortical distribution of extended sources. In COH, with two variance components accounting for minimum norm and spatial smoothness models, ReML infers the most relevant combination of these two priors to fit the data. This method was able to localize accurately spatially extended sources. We also noticed that for focal sources, COH was able to choose the minimum energy solution over the spatial smoothness solution using ReML, which indeed is a very interesting property (see Figure 2d and 4d). COH-s seemed an appropriate method for estimating spatially extended sources; the full brain parcellization of the cortical surface into non-overlapping parcels with local smoothness proved indeed to be useful. This suggested that modeling source covariance as a linear combination of covariance components and inference using ReML is an interesting methodological approach, offering sufficient flexibility in the definition of the a priori model. However, COH-s showed some instabilities in the form of spurious sources mainly in cases where the clustering scale *s* was smaller than the spatial extent *s_e_*, as well as when dealing with very focal sources (*s_e_* = 2). The method was able to find accurately the sources in most conditions but the presence of some spurious sources far from the main generators led to lower AUC and larger MSE and Dmin values. From the three proposed methods using the DDP spatial model (MEM-s, CMEM-s and COH-s), MEM-s and CMEM-s were the most accurate and stable in localizing the spatially extended sources, especially since they provided accurate results whatever was the underlying clustering scale *s*.

All approaches, except COH, presented a loss of performance when increasing the spatial extent of the source. Although, a slight decrease in the localization accuracy of MEM-s and CMEM-s was noticed for more extended sources (Figure 5c and 5d), their overall accuracy remained better than for the other methods. This decrease in the accuracy could be explained by the fact that most of the spatially extended sources (*s_e_* = 5 and 6) involved multiple sulci and gyri including substantial radial and deep components leading to more cancellation effect of the MEG signals [63]. This resulted in low amplitude MEG signal for specific regions and high amplitude for other regions (superficial and tangential components). Indeed, a spatial extension of order 5 or 6 from a superficial seed will have more chance of spreading along some mesial or basal aspects of the cortical surface, thus involving generators that are more difficult to localize. Moreover, the spatial extent *s_e_* = 5 (∼18 cm^2^) and *s_e_* = 6 (∼30 cm^2^) are less realistic (or more rare) for an expected extended epileptiform activity, whereas the extent of 4 (∼11 cm^2^) or less are the more realistically expected extents according to Huiskamp et al. (2010) [11].

### Localization of Deep Sources

Detection of deep sources is a difficult issue since deep generators will generate very low amplitude MEG data on the scalp, almost undetectable except under specific conditions. This usually requires lots of averaging to increase the SNR [64]. Within our simulation framework, we considered both deep and superficial sources, mimicking realistic SNR conditions in both cases. All the evaluated methods (except IID) accurately localized superficial sources (Figure 7), whereas they all demonstrated poor performance when localizing sub-cortical sources (eccentricity<40 mm) and some variability in accurate localization of mesio-temporal sources (40 cm<eccentricity<60 cm). Note that for eccentricity between 40 mm and 60 mm, “mesio-temporal sources” referred mainly to sources located on the mesial aspects of the temporal pole, rather than in the hippocampus per se. COH-s showed low accuracy when using small parcels (*s*<*s_e_*), even for superficial sources. On the other hand, when using larger parcels (*s*>*s_e_*) COH-s provided accurate results for superficial sources and most mesio-temporal sources. Overall, COH-s at *s* = 6 performed better than other methods when localizing sub-cortical sources in most cases of *s_e_*. It was not surprising that IID would give poor detection for deep sources because superficial dipolar sources with smaller magnitudes are favored by the minimum norm constraint. Similar trends towards most superficial sources were observed on all the evaluated methods. This bias towards the superficial sources has not been addressed in our study but few studies have been carried out to compensate such a bias [23], [29], [65]. We did not use depth weighting in any of the methods studied in this paper, in order to evaluate all the methods in the same context. However, it should be noted that even if no depth weighting was used, COH-s (for *s*>*s_e_*), MEM-s and CMEM-s and were able to localize some deeper sources (Figure 7b, 7c and 7d), more likely because the underlying model was putting forward the involvement of these parcels in the solution, similar to the Bayesian Model Averaging (BMA) approach [29].

### Spatial Models: Data Driven Parcellization and Local Smoothness Prior

The two realistic spatial models considered in this study, i.e. data driven parcellization P(s) and local spatial smoothness *W*, have been implemented within both the MEM and HB frameworks to model the activity of the underlying generators of epileptic discharges.

The first model assumed brain activity to be organized into several spatial clusters P(s) using DDP of the brain activity along the tessellated cortical surface. Few studies demonstrated how introducing parceling of the brain (obtained from some anatomical atlases) was quite useful to better condition the inverse problem [29], [31], [36], [66]. Similarly, Lapalme et al. [28] used a data driven parceling technique to partition the whole cortical surface into functionally homogenous parcels. This approach is similar to the one used in the present study. The performance of MEM-s, CMEM-s and COH-s confirms the usefulness of DDP of the whole cortical surface in detecting extended sources. Although DDP was crucial to regularize the inverse problem, the accuracy of the underlying DDP was not required to obtain an accurate MEM-s or CMEM-s localizations (Figure 6). In case of COH-s, while the size of the underlying DDP was crucial in the Hierarchical Bayesian approach, the type of data used for the parcellization did not alter the COH-s solutions. This was also confirmed with the assessment of the performance of MEM-s, CMEM-s and COH-s when the parcellization was obtained from MEG physiological background activity, instead of using the data of interest. We did not see any difference in their localization accuracy with the type of data used. We applied one method of parcellization here, but other methods could have been considered [29], [31], [36], [66], with probably similar level of localization accuracy.

However, MEM-s was unable to accurately recover the spatial smoothness of the source along its extent. This led us to incorporate the second model *W*, i.e., local spatial smoothness within the parcels, giving rise to CMEM-s method. *W* models the local spatial smoothness of the distribution of the source activity along the cortical surface. The very first idea of spatial smoothness prior was LORETA [23] that allows the smooth reconstruction of cortical sources at a low spatial resolution but does not generally reflect the focal nature of most cortical activations. *W* used in this study introduces spatial smoothness following the geodesic surface [37], thus creating local spatial smoothness within each parcel. As a result, CMEM-s was able to accurately recover the spatial smoothness of activity within the extended sources. Similarly, COH-s, which also incorporated local spatial smoothness within the parcels, was also able to recover the spatial smoothness along the source extent as soon as the size of the clustering scale *s* was larger than the extent of the source *s_e_*.

### Comparison between MEM and HB Frameworks

Two statistical regularization schemes, the MEM and the HB frameworks, were compared in this study.

COH-s method, in which we incorporated the parcellization model P(s) and the local spatial smoothness prior, was proposed to be the equivalent of CMEM-s method. It incorporates the same constraints as the CMEM-s method, but it uses the ReML algorithm within the HB framework to estimate the solution. Our findings show a good concordance between the MEM and HB frameworks when comparing the CMEM-s and COH-s for their detection accuracy, suggesting that both frameworks offer sufficient flexibility to build efficient source localization methods, especially in the context of MEG epileptic data.

In addition, we have tested the influence of the clustering scale *s* on these methods. We showed that clustering scale had no impact on detection accuracy for both MEM-s and CMEM-s, whereas COH-s method was indeed very sensitive to the clustering scale. The distribution of AUC values for COH-s shows a tendency of increase in detection accuracy when increasing the clustering scale. Indeed, we found that COH-s provided poor detection accuracy when using parcels smaller than the source spatial extent (i.e., *s*<*s_e_*), whereas for *s*>*s_e_* it provided overall good detection accuracy (see Figure 6 and 7). We could consider the highest clustering scale *s* = 6 to accurately localize the range of sources’ extents simulated in this study. However, our results still suggest that this scale parameter should be tuned from the data when localizing clinical data, as the underlying spatial extent of the generator could not be predicted.

MEM-s and CMEM-s provided very accurate results for any evaluated spatial clustering scale s. This is an important result, suggesting that MEM regularization is able to adapt the number of active parcels, whatever is the spatial scale of the clustering. In order to localize a spatially extended source as accurately as possible, MEM is able to “switch on” several parcels when using a lower clustering scale (small parcels) or only few of them when using a larger clustering scale (large parcels). Once the parcels have been identified as active, our results demonstrated that MEM inference is still able to create some local contrasts of dipole intensities within the active parcels, leading to the ability of localizing sources of different spatial extents. The regularization process was a bit different when using COH-s with ReML, as the hyper-parameters of the source covariance components (i.e., the parcels for COH-s) are first estimated through an Automatic Relevance Determination (ARD) scheme. Then, once the covariance model and its “weights” are estimated, sources are estimated using a regularized pseudo-inverse method. The diffusion-weighted prior will then push forward a spatially smooth solution over the selected parcels. When using smaller parcels with COH-s (*s*<*s_e_*) we observed that some covariance components often associated with focal sources were then falsely enhanced, leading to the occurrence of spurious sources, while the main source was still found. In addition, it is possible that when using local spatial smoothness prior within the parcels COH-s was less efficient than CMEM-s in creating contrast of dipole intensities within the parcel. However, this was out of the scope of this study and will require further investigations. Since Litvak and Friston (2008) suggested that using a Greedy Search scheme instead of ARD scheme improved the performance of Multiple Sparse Prior method [67], we can expect that using a Greedy Search for COH-s model could have similar impact on localization accuracy and stability. However, such implementation was out of the scope of the present study and will be considered for future publications. Overall, MEM framework and HB framework using ReML are two very efficient and sufficiently flexible regularization approaches that can be adapted to localize spatially extended sources. An important aspect of ReML-based approaches is that quantitative model comparison using free energy [26] is possible and may be quite useful when testing the relevance of several models, as demonstrated in Henson et al. (2009) [45].

Note that, in this work, we compared the performances of the five methods using the same forward model for both source simulation and source localization. This is a standard approach when assessing the performances of source localization methods [16], [27], [29], [31], [58], [68]. This is a best-case scenario and a decrease in performance should be expected when applied on real data. However, we investigated the detection accuracy of all the methods when using either the same forward model for both simulation and localization (1 layer BEM model) or a BEM model for simulation and an analytical spherical model for localization (results not shown). All the inverse solvers then outperformed in the same proportion when different models were used, without modifying the overall relative performances. We can conclude that our evaluation did not bias the results of any particular method, allowing appropriate comparison between methods, which was the purpose of this study.

### Practical Application and Future Work

In distributed source modeling, the cortical surface constraint is defined from large cortical assemblies of pyramidal cells organized orthogonally to the grey-white matter interface. Most distributed methods adopt this constraint with either restricting the orientation to be perpendicular [21] or allowing some deviation from the surface normal [69]. Lin et al. (2006) [69] and Henson et al. (2009) [45] showed that the use of loose orientation dipolar sources increased the localization accuracy of minimum norm methods. In our distributed model, although we fixed the orientation normal to the surface, the loose orientation constraint could be easily adapted and would impact all the methods similarly; but this falls out of scope of the present study.

When dealing with real data, it is important to study the impact of the quality of segmentation and resolution of the cortical surface on the source reconstruction, and especially in pathological conditions. On the other hand, Henson et al. (2009) [45] showed that the nature of the cortical surface (obtained from individual MRI or warping the individual’s MRI to a template MRI) had only minimal impact on MEG source reconstruction, as soon as the overall head model was transformed to take into account the actual head shape of the subject. They also showed that the resolution of the cortical surface (3000 sources or 7000 sources) had no reliable effect on the Minimum norm solution; whereas the 7000 sources surface resulted in better accuracy for their proposed Multiple Sparse Prior model. Whereas these are important issues to cope with, they will have similar influence on all the methods involved in the present evaluation study.

Our simulation paradigm was a spatial validation, studying detection accuracy only at the main peak of the simulated spike. In our future work, we plan to assess the spatio-temporal features of the sources by simulating different propagation patterns of epileptic discharges using models such as the extended source model developed by Cosandier-Rimélé et al. [53], [70], which describes both the spatial distribution and the temporal dynamics of neuronal population. The code for MEM-s and CMEM-s methods has been recently implemented as a toolbox in Brainstorm Package [71] (http://neuroimage.usc.edu/brainstorm/), to be released soon.

### Conclusion

We have proposed three new methods (MEM-s, CMEM-s and COH-s) and evaluated their performance when localizing spatially extended generators of epileptic discharges using MEG data. We demonstrated that modeling brain activity using Data Driven Parcellization of the cortical surface and applying local smoothness within each parcel is particularly relevant to localize sources together with their spatial extent. Both MEM and HB frameworks are sufficiently flexible to allow the implementation of such spatial models. The present study is in agreement with the good performance of MEM we previously demonstrated on EEG data, although we added the evaluation of several other parameters such as a larger range of spatial extents and depths of the sources, as well as the scale of the spatial clustering.

## Supporting Information

Appendix S1 **Spatio-temporal extension of the Multivariate Source Pre-localization (MSP).**
(DOCX)

Appendix S2 **Data Driven Parcellization (DDP).**
(DOCX)

Appendix S3 **Maximum Entropy on the Mean (MEM) formulation.**
(DOCX)

Appendix S4 **Spatio-temporal initialization of** ***α_k_*** **.**
(DOCX)
