# Supplementary material for: MEG Source Localization of Spatially Extended Generators of Epileptic Activity: Comparing Entropic and Hierarchical Bayesian Approaches
Source: PLoS One. 2013 Feb 13;8(2):e55969. doi: 10.1371/journal.pone.0055969 (PMC3572141; doi:10.1371/journal.pone.0055969)
Supplement: Appendix S1 — Spatio-temporal extension of the Multivariate Source Pre-localization (MSP). (DOCX) [file pone.0055969.s001.docx]

**Spatio-temporal extension of the Multivariate Source Pre-localization (MSP)**

The MSP method proposed by Mattout et al. (2005) aims at estimating the contribution of each dipolar source to the data. This is obtained by exploiting the MEG normalized data $(\overline{M})$ and the normalized lead field matrix$(\overline{G})$ (normalization by the norm of each column).

In order to account for the spatio-temporal behavior of the brain activity, we used a Singular Value Decomposition (SVD) of the data matrix *M*. This extracts the most meaningful components and their corresponding time courses. The SVD of *M* is expressed as:

$M= U\Lambda V^{T}$ (S.1)

where, *U* is a ($\text{q}\times\text{q})$ orthogonal matrix, in which the $l^{th}$ column vector is the sensor signature of the $l^{th}$ component. $\Lambda$ is a $(\text{q}\times\text{τ})\text{ }$ matrix whose diagonal contains the singular values of *M* and *V* is the $(\text{τ}\times\text{τ})$ orthogonal matrix containing the time courses associated to each component. We identified the signal subspace by keeping the first $c^{*}$ principal components accounting for 95% of the total variance. For each selected principal component $l$, we obtained dual information i.e., the time courses $V_{l}$ and sensor signatures of the sources likely to follow such a time course$U_{l}$. The remaining data subspace defines the noise subspace denoted by $M_{n}$(n=q–c^*^ being the dimension of the noise subspace).

**Estimating the contribution of the dipoles to each principal component**: To estimate the contribution of each dipolar source to each principal component $U_{l}$ ($l\in\left[1, c^{*}\right]$), we used the MSP, which assigns to each source a probability like coefficient of activation .

These coefficients are quantified by projecting the normalized lead field $\overline{G}$ onto the normalized data $U_{l}$ as below:

$\xi=\left(\overline{G}^{T}U_{l}U_{l}^{T}\overline{G}\right)$, (S.2)

The norm of the projection of normalized lead field, quantifies the correlation between the contribution of the dipoles to the forward field and the data of interest. The diagonal of$\xi$ defines a $\text{p}\times1$ column vector containing the probability-like coefficients of activation associated with the $l^{th}$ principal component. These $c^{*}$columns vectors for $l\in[1, c^{*}]$ define matrix$B=[b_{il}]$, which represents the contribution of each dipole *i* to $l^{th}$ principal component. A higher probability coefficient denotes higher correlation between the corresponding dipole and the data.
