# Supplementary material for: MEG Source Localization of Spatially Extended Generators of Epileptic Activity: Comparing Entropic and Hierarchical Bayesian Approaches
Source: PLoS One. 2013 Feb 13;8(2):e55969. doi: 10.1371/journal.pone.0055969 (PMC3572141; doi:10.1371/journal.pone.0055969)
Supplement: Appendix S2 — Data Driven Parcellization (DDP). (DOCX) [file pone.0055969.s002.docx]

**Data Driven Parcellization (DDP)**

***Definition of the seed points:*** This step consisted in selecting K seed points uniformly distributed over the whole cortical surface. The total number of seed points K will then tune the spatial extent of the parcels, thus the spatial scale *s* of the partition P($\text{s}$). Let us denote $b_{il}$ (from APPENDIX S1), the MSP coefficients of the dipole *i* for the component *l* ($l\in\left[1, c^{*}\right]$). We first define pre-clusters by assigning each dipole *i* to the component *l** corresponding to its maximum contribution $b_{il}$ for$l\in\left[1, c^{*}\right]$. Within each of these regions, i.e, each pre-cluster assigned to a component *l*, we identified seed points iteratively:

- The first seed point corresponds to the dipole *i_1_* showing the maximum MSP value $b_{il}$
- All dipoles belonging to the $\text{s}^{th}$ neighborhood order[[1]](#footnote-1) of that seed point are removed from the list of potential seed points.
- Among the remaining potential seed points, assigned to the $l^{th}$ pre-cluster, the dipole *i_2_* with the next highest MSP value $b_{il}$ is selected as the second seed point.
- The process is iterated until all dipoles of $l^{th}$pre-cluster have been considered.

To achieve a complete parceling of the brain, this iterative process is done sequentially for each selected component$l\in\left[1, c^{*}\right]$ and then finally within the remaining noise subspace.

***Region growing around the seed points:*** Spatial clustering was then completed using a region growing algorithm around each of the K seed points defined in the previous section. Region growing along the geodesic cortical surface was constrained to ensure that dipoles assigned to distinct components *l* and *l’* (*l* and *l’* $\in\left[1, c^{*}\right]$) were not included in the same parcel. A particular spatial clustering scale *s* will actually correspond to the average number of iterations of the region growing around the seed points, leading to a total of K parcels.

1. Neighborhood order refers to spatial connections of dipoles along the geodesic cortical surface. 1^st^ neighborhood order refers to dipoles directly connected on the mesh, and so on. [↑](#footnote-ref-1)
