# Supplementary material for: MEG Source Localization of Spatially Extended Generators of Epileptic Activity: Comparing Entropic and Hierarchical Bayesian Approaches
Source: PLoS One. 2013 Feb 13;8(2):e55969. doi: 10.1371/journal.pone.0055969 (PMC3572141; doi:10.1371/journal.pone.0055969)
Supplement: Appendix S3 — Maximum Entropy on the Mean (MEM) formulation. (DOCX) [file pone.0055969.s003.docx]

**Maximum Entropy on the Mean (MEM) formulation**

Given the generative model of data M in equation 1 and considering the reference distribution $d\nu(j)$is initialized as in equation 5 and 6, the MEM solution $d\hat{p}$is defined by:

$d\hat{p}\left(j\right)= \frac{e^{\tilde{\beta}^{T}Gj}}{Z\left(\tilde{\beta}\right)}d\nu(j)$ (S.3)

where $\tilde{\beta}$ is the maximum of the non-linear optimization of a convex function in a q-dimensional space (see for the proof), thus giving a unique solution

$\tilde{\beta}=argmax_{\beta}(\beta^{T}M- F_{\nu}\left(G^{T}\beta\right)- \frac{\sigma_{E}^{2}}{2}\beta^{T}\beta)$ (S.4)

and $Z\left(\tilde{\beta}\right)= e^{F_{\nu}\left(G^{T}\tilde{\beta}\right)}$ with $F_{\nu}\left(G^{T}\tilde{\beta}\right)$ as the “free energy” term associated to $d\nu$

$F_{\nu}\left(G^{T}\tilde{\beta}\right)= \log\int^{ } \text{ e}^{\tilde{\beta}^{T}Gj}d\nu(j)$ (S.5)

and $\sigma_{E}$ is the covariance of *E* in equation 1.

It can then be shown that the MEM estimate of the sources’ intensities *J* could be obtained from the gradient of the free energy.

$\hat{J}_{MEM}= \nabla_{\text{s}}\text{F}_{\nu}\left(\text{s}\right)|_{\text{ s}=G^{T}\tilde{\beta}}$ (S.6)

Considering the above formulations, the MEM estimate of the sources in each parcel *k* can be found to be: $\hat{J}_{MEM}^{k}= \hat{\alpha}_{k}[\mu_{k}+\sum_{k}G_{k}^{T}\tilde{\beta}]$ (S.7) $\hat{\alpha}_{k}=\frac{\alpha_{k}}{\alpha_{k}+(1-\alpha_{k})\exp(-F_{\nu,k}(G_{k}^{T}\tilde{\beta}))}$ (S.8)

Where $F_{\nu,k}$ is the free energy corresponding to the *k^th^* parcel when active (i.e., $S_{k}$ = 1), given by:

$F_{\nu,k}\left(G_{k}^{T}\tilde{\beta}\right)= \mu_{k}^{T}G_{k}^{T}\tilde{\beta}+\frac{1}{2}\tilde{\beta}^{T}G_{k}\sum_{k}G_{k}^{T}\tilde{\beta}$ (S.9)

and $G_{k}$is the $(q\times p_{k})$submatrix of *G*.
