# Supplementary material for: MEG Source Localization of Spatially Extended Generators of Epileptic Activity: Comparing Entropic and Hierarchical Bayesian Approaches
Source: PLoS One. 2013 Feb 13;8(2):e55969. doi: 10.1371/journal.pone.0055969 (PMC3572141; doi:10.1371/journal.pone.0055969)
Supplement: Appendix S4 — Spatio-temporal initialization of αk . (DOCX) [file pone.0055969.s004.docx]

**Spatio-temporal initialization of** $\alpha_{k}$

For each cortical parcel k, the spatio-temporal behavior of activity $(\alpha_{k})$ was estimated using a “spatio-temporal” activation probability map (stAPM) defined as:

$stAPM(i,t)= \frac{1}{max} \sum_{l=1}^{l=c*} b_{il}|v_{tl}|$ (S.10)

where $b_{il}$contains the MSP coefficients of the dipole *i* corresponding to the $l^{th}$ principal component and $v_{tl}$ represents the time sample associated with the $l^{th}$column of $V$(see equation S.1 in APPENDIX S1), $max$ is a normalization factor with respect to time ensuring that the resulting coefficients belongs to [0,1]. At each time sample$t$, the probability of (the set of $p_{k}$ sources in the $\text{k}^{\text{th}}$parcel) to be active was defined as the median value along the parcel.
